# Supplementary material for: Zn and Zn-Fe Nanostructures with Multifunctional Properties as Components for Food Packaging Materials
Source: Nanomaterials (Basel). 2022 Jun 18;12(12):2104. doi: 10.3390/nano12122104 (PMC9230730; doi:10.3390/nano12122104)
Supplement: Supplementary file 1 [file nanomaterials-12-02104-s001.zip › nanomaterials-1773004-supplementary.pdf]

nanomaterials

Supplementary Material

Zn and Zn-Fe Nanostructures with Multifunctional Properties as Components for Food Packaging Materials

Hafsae Lamsaf [1,2], Lina F. Ballesteros [3,4], Miguel A. Cerqueira [2], José A. Teixeira [3,4], Lorenzo M. Pastrana [2], Luís Rebouta [1], Sandra Carvalho [5] and Sebastian Calderon V [1,2,*]

[1] CF-UM-UP, Centre of Physics of Minho and Porto Universities, Campus of Azurém, 4800-058 Guimarães, Portugal; hafsae.lamsaf@gmail.com (H.L.); rebouta@fisica.uminho.pt (L.R.)
[2] INL—International Iberian Nanotechnology Laboratory, Av. Mestre José Veiga s/n, 4715-330 Braga, Portugal; miguel.cerqueira@inl.int (M.A.C.); lorenzo.pastrana@inl.int (L.M.P.)
[3] CEB—Centre of Biological Engineering, University of Minho, Campus of Gualtar, 4710-057 Braga, Portugal; linafernanda37@ceb.uminho.pt (L.F.B.); jateixeira@deb.uminho.pt (J.A.T.)
[4] LABBELS–Associate Laboratory, Braga/Guimarães, Portugal
[5] CEMMPRE, Mechanical Engineering Department, University of Coimbra, 3030-788 Coimbra, Portugal; sandra.carvalho@dem.uc.pt
* Correspondence: secave44@gmail.com

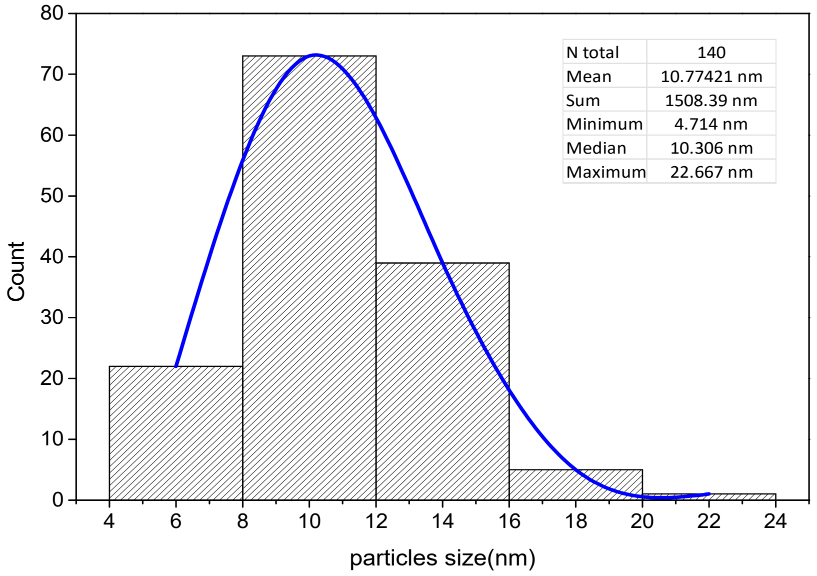

Figure S1. Size distribution of Fe particles in Zn NLs+Fe NPs sample.

The ratio of Zn/Fe samples obtained by ICP and EDS for each sample is presented in Table S1. The Zn/Fe values are quite similar for Zn-Fe alloy and Zn-Fe NLs coatings, and it is expected to be higher for Zn NLs + Fe NPs since the coating is deposited on a centered line of 3 mm surrounded by only Zn NPs (see the real image of the Zn NLs + Fe NPs sample in Figure S2).

Table S1. Values of Zn/Fe ratios obtained by ICP and EDS.

Coating	Zn/Fe ratio	
	ICP-OES	EDS
ZnFe alloy	2.5	1.91
ZnNLs + FeNPs	34.14	10.12
ZnFe NLs	18.34	12.71

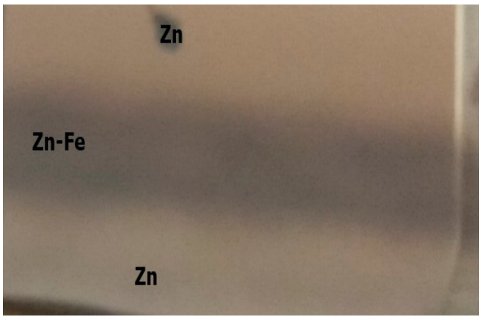

Figure S2. Real image of Zn NLs + Fe NPs coating.
